# Assessing the effects of Cry1C rice and Cry2A rice to *Pseudogonatopus flavifemur*, a parasitoid of rice planthoppers

**DOI:** 10.1038/s41598-017-08173-w

**Published:** 2017-08-10

**Authors:** Jun-Ce Tian, Jörg Romeis, Kai Liu, Fa-Cheng Zhang, Xu-Song Zheng, Hong-Xing Xu, Gui-Hua Chen, Xiao-Chan He, Zhong-Xian Lu

**Affiliations:** 1 Key Laboratory of Information Traceability for Agricultural Products, Ministry of Agriculture of China, Institute of Plant Protection and Microbiology, Zhejiang Academy of Agricultural Sciences, 198 Shiqiao Road, Hangzhou, 310021 China; 2 Agroscope, 8046 Zurich, Switzerland; 3 Jinhua Plant Protection Station, Jinhua, 321017 China; 4 Jinhua Research Academy of Agricultural Sciences, Jinhua, 321017 China

## Abstract

Transgenic rice producing insecticidal proteins from *Bacillus thuringiensis* (Bt) could help protect the plants from damage by lepidopteran pests. However, one concern is the potential of Bt rice to harm non-target natural enemies, which play a vital role in pest control. In the present study, the potential effects of Cry1C rice and Cry2A rice on different life-table parameters and population dynamics of *Pseudogonatopus flavifemur*, a parasitoid of rice planthoppers, were evaluated under laboratory and field condition. The exposure of *P*. *flavifemur* to plant-produced Bt proteins was also analyzed. Results indicated that direct feeding on rice plants was the main exposure pathway of *P*. *flavifemur* to the Cry1C and Cry2A proteins. No significant difference on the development, survival, longevity, fecundity, and prey consumption of *P*. *flavifemur* was detected over two generations between the Bt and non-Bt rice treatments. Furthermore, the population dynamics of *P*. *flavifemur* were not affected by Cry1C rice and Cry2A rice. In conclusion, the tested Cry1C rice and Cry2A rice do not appear to harm the parasitoid *P*. *flavifemur*.

## Introduction

Rice, *Oryza sativa* L., is one of the principal staple foods in the world. More than 50% of the world populations depend on rice for their daily lives^1^. According to various estimates, the global population is expected to reach 9.0 billion by 2050^2^, and 40% more rice must be produced to meet the increasing needs of the projected human population; therefore, improvements in rice yields are urgently required. However, 13–26% of rice yield are lost due to pests^3^. Rice stem borers, for example, are responsible for 3–10% annual loss in yield and economic losses of 11.5 billion yuan ($US 1.85 billion) annually in China alone^4^. Numerous genetically modified (GM) rice lines expressing insecticidal crystal (Cry) proteins from *Bacillus thuringiensis* Berliner (Bt) have been developed to control Lepidoptera pests, i.e., stem borers and leaffolders^5, 6^. Field studies with a Bt rice line in China revealed an increase in yield by 6–9% and a reduction in pesticides usage by 80%^7^. Due to those potential benefits, the Chinese government has issued the biosafety certificates and approved limited releases of two Cry1Ab/Cry1Ac rice lines in farmers’ fields in Hubei Province from 2009 to 2014^8^ with an extension from 2014–2019^9^.

However, before being used widely the impact of Bt rice on the environment should be assessed^10, 11^. One concern is the potential of Bt rice to adversely affect natural enemies which play a vital role in pest control^11^. To date, a series of studies have focused on the impacts of Bt rice on the population dynamics, abundance and diversity of natural enemies^12–16^. In addition, laboratory studies have been conducted to assess the impact of Bt rice on lethal and sublethal endpoints of important natural enemy species that are common in Chinese rice fields (Li *et al*. 2017). These include the predators *Cyrtorhinus lividipennis* (Hemiptera: Miridae)^17–19^, *Propylea japonica* (Coleoptera: Coccinellidae)^20, 21^, *Paederus fuscipes* (Coleoptera: Staphylinidae)^22^, *Chrysoperla nipponensis* (as *Chrysoperla sinica*) (Neuroptera: Chrysopidae)^23^, *Ummeliata insecticeps* (Araneida: Linyphiidae)^24^, *Pardosa pseudoannulata* (Araneae: Lycosidae)^25^, and the parasitoid *Anagrus nilaparvatae* (Hymenoptera: Mymaridae)^26, 27^. However, the risks of Bt rice on parasitoids attacking planthoppers that are not affected by the insecticidal trait have not been assessed so far.

*Pseudogonatopus flavifemur* (Hymenoptera: Dryinidae) is one of the most common parasitoids of *Nilaparvata lugens* (Hemiptera: Delphacidae)^28^, which is one of the most serious rice pests in south Asian, and is consistently characterized by a sexual dimorphism^29^. The ant-like, wingless female wasps deposit their eggs on nymphs and adults of *N*. *lugens* in addition to host feeding on the planthoppers^29^. The wasps could thus be exposed to Bt proteins when larvae or female adults feed on Bt rice-fed *N*. *lugens*. In the present study, we evaluated the exprosure pathways of *P*. *flavifemur* to Cry1C and Cry2A produced by Bt rice, the tri-trophic effects of Bt rice on different life-table parameters of *P*. *flavifemur* and the potential effects of Bt rice on *P*. *flavifemur* populations in the field.

## Results

### Tri-trophic bioassay with *P*. *flavifemur*

Studies were conducted to assess the impact of Bt rice feeding by *N*. *lugens* on the performance of the parasitoid *P*. *flavifemur*. Eight to eleven days after parasitism, *P*. *flavifemur* larvae formed cocoons and adults emerged 10.5–14 days later. For both parasitoid generations studied, no significant differences among the two Bt and the non-Bt rice lines were detected on a number of important life-table parameters of *P*. *flavimur* (Table 1).

**Table 1:** Tri-trophic effects on life-table parameters (mean ± SE) of *Pseudogonatopus flavifemur* when provided *Nilaparvata lugens* nymphs that were reared on Cry1C, Cry2A or non-Bt rice plants over two generations.

Parameters	Cry1C rice	Cry2A rice	Non-Bt rice	Statistics
1^st^ generation				
Development (days)				
Eggs to cocoons	9.7 ± 0.3	9.6 ± 0.4	9.4 ± 0.4	*F* = 0.14; df = 2, 29; *P* = 0.87
Male eggs to adults	22.3 ± 0.4	22.2 ± 0.2	22.0 ± 0.3	*F* = 0.14; df = 2, 29; *P* = 0.87
Female eggs to adults	23.0 ± 0.5	22.7 ± 0.3	22.8 ± 0.5	*F* = 0.12; df = 2, 29; *P* = 0.89
Cocoon to adults survival (%)	84.7 ± 1.7	83.7 ± 2.2	81.6 ± 2.5	*F* = 0.52; df = 2, 29; *P* = 0.60
Male longevity (days)	2.7 ± 0.2	3.1 ± 0.3	2.9 ± 0.3	*F* = 0.53; df = 2, 29; *P* = 0.59
Female longevity (days)	11.3 ± 1.5	10.1 ± 1.2	10.4 ± 1.6	*F* = 0.17; df = 2, 29; *P* = 0.84
No. consumed nymphs	47.9 ± 5.3	45.6 ± 6.5	43.9 ± 3.6	*F* = 0.14; df = 2, 29; *P* = 0.87
Fecundity	53.0 ± 6.9	49.4 ± 6.2	51.3 ± 5.7	*F* = 0.08; df = 2, 29; *P* = 0.92
Sex raito (%)	33.3 ± 1.6	32.2 ± 1.7	31.7 ± 1.5	*F* = 0.27; df = 2, 29; *P* = 0.76
2^nd^ generation				
Development (days)				
Eggs to cocoons	9.8 ± 0.3	9.6 ± 0.3	9.5 ± 0.3	*F* = 0.40; df = 2, 29; *P* = 0.67
Male eggs to adults	22.5 ± 0.3	22.0 ± 0.2	22.4 ± 0.3	*F* = 0.20; df = 2, 29; *P* = 0.80
Female eggs to adults	23.2 ± 0.4	23.1 ± 0.2	23.4 ± 0.4	*F* = 1.04; df = 2, 29; *P* = 0.37
Cocoon to adults survival (%)	79.5 ± 2.1	76.0 ± 1.3	78.5 ± 2.6	*F* = 0.55; df = 2, 29; *P* = 0.59
Male longevity (days)	3.1 ± 0.3	3.1 ± 0.3	3.0 ± 0.3	*F* = 0.08; df = 2, 29; *P* = 0.92
Female longevity (days)	9.3 ± 1.1	9.0 ± 0.8	9.9 ± 1.4	*F* = 0.15; df = 2, 29; *P* = 0.86
No. consumed nymphs	48.0 ± 7.2	46.8 ± 5.1	50.2 ± 7.9	*F* = 0.05; df = 2, 29; *P* = 0.96
Fecundity	45.6 ± 5.4	48.4 ± 2.9	43.7 ± 3.9	*F* = 0.31; df = 2, 29; *P* = 0.74
Sex ratio (%)	30.7 ± 2.2	29.3 ± 2.1	31.8 ± 2.0	*F* = 0.25; df = 2, 29; *P* = 0.79

Ten replications were tested for each treatment. No significant difference was found among treatments based on one-way ANOVA (*P* < 0.05).

### Bt protein levels in rice plants, *N*. *lugens* and *P*. *flavifemur*

Stems of Cry1C rice contained a mean of 3.86 μg/g fresh weight (FW) (Table 2). The average of Cry1C protein detected in *P*. *flavifemur* that had been exposed to Bt rice plants infested with *N*. *lugens* or to uninfested Bt rice plants for 48 h was 0.15 μg/g and 0.143 μg/g FW, respectively, which was significantly lower than those in Cry1C rice stem, but significantly higher than those in *N*. *lugens* (0.053 μg/g FW) (*F* = 225.82; df = 3, 19; *P* < 0.001).

**Table 2:** Bt protein levels in Bt rice plants, *Nilaparvata lugens* and *Pseudogonatopus flavifemur*.

Sample	Amount (μg/g FW)		
	Cry1C rice	Cry2A rice	non-Bt rice
Rice stem	3.86 ± 0.50 a	9.07 ± 0.44 a	n.d.
*N*. *lugens*	0.053 ± 0.008 c	0.067 ± 0.007 c	n.d.
*P*. *flavifemur* larvae	n.d.	n.d.	n.d.
*P*. *flavifemur* cocoons	n.d.	n.d.	n.d.
Newly emerged male *P*. *flavifemur*	n.d.	n.d.	n.d.
Newly emerged female *P*. *flavifemur*	n.d.	n.d.	n.d.
*P*. *flavifemur* exposed to Bt rice infested with *N*. *lugens*	0.150 ± 0.010 b	0.275 ± 0.022 b	n.d.
*P*. *flavifemur* exposed to uninfested Bt rice	0.143 ± 0.007 b	0.236 ± 0.028 b	n.d.

Means (±SE) within a column followed by different letters are significantly different (One-way ANOVA, *P* < 0.05); N = 5. n.d. – not detectable. The detection limit for the two Cry proteins was 1 ng/g.

Similar results were found for the Cry2A rice. The Cry2A protein levels in *P*. *flavifemur* exposed to Bt rice infested with *N*. *lugens* (0.28 μg/g FW) or to uninfested Bt rice (0.24 μg/g FW) were significantly lower than those in Cry2A rice stems (9.07 μg/g FW), but significantly higher than those in *N*. *lugens* (0.067 μg/g FW) (*F* = 304.61; df = 3, 19; *P* < 0.001).

As expected, no Bt protein was detected in the stem, *N*. *lugens* and *P*. *flavifemur* from the respective non-Bt rice treatment.

### *P*. *flavifemur* and *N*. *lugens* populations in Bt rice and non-Bt rice fields

**Figure 1:**
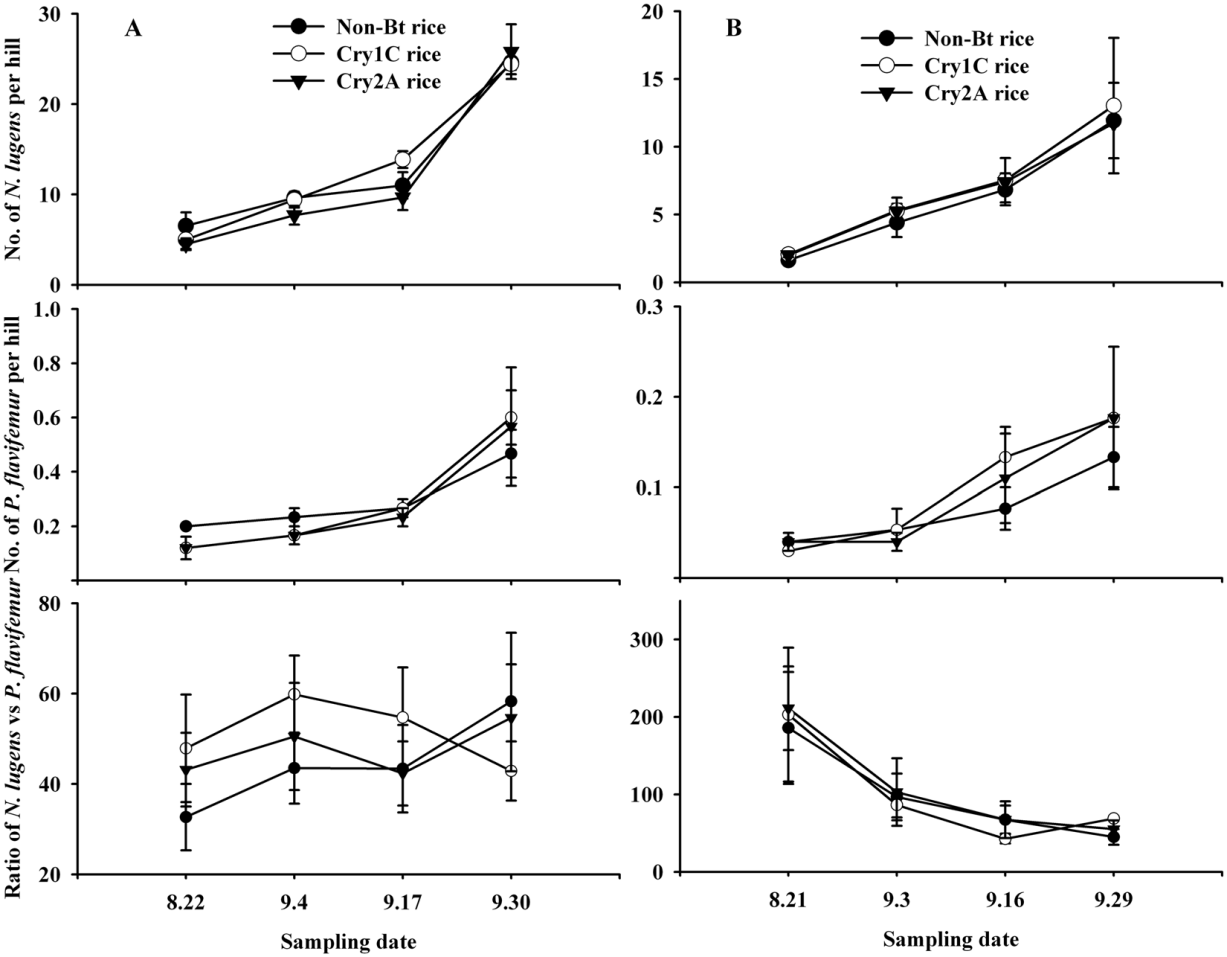
Population dynamics of *Nilaparvata lugens* and *Pseudogonatopus flavifemur* in 2013. Data are represented as mean ± SE. (**A**) Dongyan field site; (**B**) Jinha field site. There was no significant difference between the Cry1C, Cry2A and non-Bt rice fields (repeated-measured ANOVA and Tukey’s multiple comparison tests, *P* < 0.05).

Field experiments were conducted at two experimental sites. At the Dongyang site, sampling date significantly affected the populations of *N*. *lugens* (*F* = 118.80; df = 3, 35; *P* < 0.001) and *P*. *flavifemur* (*F* = 34.73; df = 3, 35; *P* < 0.001). The factor rice line (*N*. *lugens*: *F* = 0.97; df = 2, 35; *P* = 0.39; *P*. *flavifemur*: *F* = 0.17; df = 2, 35; *P* = 0.85) and the interaction between sampling date and rice line were not significant (*N*. *lugens*: *F* = 1.01; df = 6, 35; *P* = 0.45; *P*. *flavifemur*: *F* = 0.44; df = 6, 35; *P* = 0.84). The ratio of *N*. *lugens* to *P*. *flavifemur* was not significantly effect by rice line (*F* = 0.73; df = 2, 35; *P* = 0.52) or sampling date (*F* = 1.14; df = 3, 35; *P* = 0.41) and the interaction between the two factors was not significant (*F* = 0.63; df = 6, 35; *P* = 0.70) (Fig. 1A).

Similar results were found at the Jinhua site. Although sampling date significantly affected the populations of *N*. *lugens* (*F* = 13.40; df = 3, 35; *P* < 0.001) and *P*. *flavifemur* (*F* = 6.33; df = 3, 35; *P* = 0.003), the populations did not differ among rice lines (*N*. *lugens*: *F* = 0.15; df = 2, 35; *P* = 0.87; *P*. *flavifemur*: *F* = 0.34; df = 2, 35; *P* = 0.72) and the interaction between sampling date and rice line were not significant (*N*. *lugens*: *F* = 0.03; df = 6, 35; *P* = 0.99; *P*. *flavifemur*: *F* = 0.21; df = 6, 35; *P* = 0.97). For the ratio of *N*. *lugens* to *P*. *flavifemur*, sampling date significantly affect the ratio (*F* = 8.21; df = 2, 35; *P* < 0.001), but rice line did not (*F* = 0.08; df = 2, 35; *P* = 0.92) and the interaction between sampling date and rice line was also not significant (*F* = 0.09; df = 6, 35; *P* = 0.99) (Fig. 1B).

## Discussion

Natural enemies of crop pests may be at risk from the growing of Bt rice if they are exposed to the insecticidal Cry proteins when attacking their prey or hosts. That the Bt rice produced Cry proteins move through the arthropod food web in rice has recently been confirmed^28, 30^. In the present study, we confirmed the presence of Cry1C and Cry2A in the tissue of two Bt rice lines and in *N*. *lugens* that had fed on the plants, albeit at a very low concentration (reduced by a factor of 72 and 135 when compared to the two Bt rice lines, respectively). No Bt protein was detected in *P*. *flavifemur* larvae, cocoons, and newly emerged adults that had developed in Bt rice-fed *N*. *lugens*. Similar results were reported from other tri-trophic studies involving a Bt plant, a herbivore and a parasitoid. When *Cotesia marginiventris* (Hymenoptera: Braconidae) developed in Cry1Ac maize-fed *Spodoptera littoralis* ^31^ or in Cry1F maize-fed *Spodoptera frugiperda* (both Lepidoptera: Noctuidae)^32^, Bt protein levels in *C*. *marginiventris* larvae, cocoons, and adults were below the detection limit. In the case of *P*. *flavifemur*, however, female wasps were found to contain Bt protein when being exposed to Bt rice plants infested with *N*. *lugens*. The Bt protein levels in female wasps were significantly lower than those in Bt rice (by a factor of 25) but significantly higher (by a factor of 3) that those detected in *N*. *lugens*. Previous studies conducted with predators of *N*. *lugens* such as the spiders *U*. *insecticeps* ^24^ and *P*. *pseudoannulata* ^25^ and the rove beetle *P*. *fuscipes* had revealed significantly lower Bt levels in the predators when compared to those in the prey, i.e., Bt rice-fed *N*. *lugens*. Thus, we conducted an additional experiment to test the Bt protein levels in *P*. *flavifemur* that were exposed to Bt rice alone. The fact that we were able to detect similarly high amounts of Bt proteins in the wasps indicates that they must have consumed plant material, a fact that has not been reported before. *P*. *flavifemur*, as many other species of Dryinidae, are known to possess strong mandibles that allow them to bite the host’s integument in order to feed on the leaking haemolymph^33^. It is thus possible that they are able to also feed on the plants directly. Consequently, feeding on the rice plants rather than host feeding on *N*. *lugens* appears to be their main pathway of exposure. A similar finding has been reported for an omnivorous predator, the mirid bug *C*. *lividipennis*. The Cry2Aa protein levels in *C*. *lividipennis* that had been provided with Cry2Aa rice plants was higher than those that had consumed Cry2Aa rice-fed *N*. *lugens* ^19^.

Though female *P*. *flavifemur* adults were exposed to Cry1C or Cry2A proteins in our tri-trophic bioassays, no significant difference in development, survival, longevity, fecundity, prey consumption, and progeny sex ratio were found between Bt rice and non-Bt rice lines over two generations.

The current study is the first to assess the potential effects of Bt rice on a dryinid wasp. Our results are in accordance with those obtained previously for the same Bt rice lines containing Cry1C or Cry2A or purified Cry proteins and different natural enemies. Larvae of the green lacewing *Chrysoperla japonica* (as *C*. *sinica*) (Neuroptera: Chrysopidae) or the ladybird beetle *P*. *japonica* where not affected by Cry1C or Cry2A when fed pollen from Bt rice or high doses of purified Bt protein provided within artificial diet^23, 34^. Similarly, *C*. *lividipennis* ^19^ and *Hylyphantes graminicola* (Araneae: Linyphiidae)^35^ were not adversely affected when feeding Cry2A rice-fed *N*. *lugens*. One study was conducted with a parasitoid, *A*. *nilaparvatae*. The authors reported no difference in survival, development, longevity, and fecundity of *A*. *nilaparvatae* that had developed in eggs of *N*. *lugens* reared on Cry2A rice from those emerging from eggs on non-Bt rice^26^.

To support the non-target risk assessment of Bt crops, early-tier laboratory experiments should be conducted under worst-case exposure condition^11, 36^. Our study, however, was conducted at realistic exposure levels. We have thus also conducted field experiments to assess the impact of Cry1C rice and Cry2A rice on *P*. *flavifemur* populations. Our two-site field experiments showed that Cry1C rice and Cry2A rice did not affect the populations of *N*. *lugens* and *P*. *flavifemur* as well as the ratio of *N*. *lugens* to *P*. *flavifemur*. These results are in accordance with a number of studies that have not seen any effect of Cry1C rice and Cry2A rice on the population dynamics of *Nephotettix cincticeps* (Hemiptera: Cicadellidae)^37^, as well as planthoppers (*N*. *lugens*, *Sogatella furcifera* and *Laodelphax striatellus*) (Homoptera: Delphacidae) and their predators [*C*. *lividipennis*, *Pirata subpiraticus* (Araneae: Lycosidae) and *Theridium octomaculatum* (Araneae: Theridiidae)]^12^. Similarly, the population of *H*. *graminicola*, a generalist predator of *N*. *lugens*, was not affected by Cry2A rice^35^. All the field results are thus consistent with the results from the laboratory study, which indicated the biosafety of Cry1C rice and Cry2A rice on non-target arthropod^28^.

In summary, *P*. *flavifemur* was exposed to Bt proteins by directly feeding on Bt rice plants rather than through their hosts. However, the tested Cry1C rice and Cry2A rice lines did neither affect the development, survival, longevity, fecundity, and prey consumption of *P*. *flavifemur*, nor their population dynamic in the field.

## Methods

### Plants

Two transgenic Bt rice lines, T1C-19 (Cry1C rice) expressing Cry1C protein and T2A-1 (Cry2A rice) expressing the Cry2A protein, and the untransformed parental commercial non-Bt rice MH63 were used for laboratory and field evaluation. The gene *cry1C* and *cry2A* gene were synthesised on the basis of the amino acid sequence of the corresponding wild-type *cry1Ca5* gene and *cry2Aa* gene of *B*. *thuringiensis* and both driven by maize ubiquitin promoter^38, 39^. Both transgenic Bt rice lines have high resistance to stem borers and leaffolders under laboratory and field conditions^40, 41^. MH63 is an elite *indica* restorer strain for cytoplasmic male-sterility in China and served as the control. All the above rice lines were supplied by the National Key Laboratory of Crop Genetic Improvement and National Centre of Plant Gene Research (Wuhan), Huazhong Agricultural University, China. Taichung Native 1 (TN1), a pest-susceptible rice variety obtained from the International Rice Research Institute (Los Baños, Laguna, Philippines), was used to maintain the *N*. *lugens* colony.

The rice plants were cultured in a plastic tank (200 cm length × 50 cm width × 15 cm height) in Yoshida culture solution^42^ in the greenhouse. 45-day-old rice seedlings were used in the laboratory experiments. All the plants were maintained at 26 ± 2 °C and the relative humidity was 75 ± 5%.

### Insects

A colony of *N*. *lugens* was collected from paddy fields (30.31° N, 120.19° E) in the suburb of Hangzhou, Zhejiang Province, China, and maintained on TN1 at 26 ± 2 °C, 75 ± 5% RH, under a light and dark regime of 14:10 h. Prior to the tri-trophic bioassays, independent colonies of *N*. *lugens* were established on Cry1C rice, Cry2A rice and non-Bt rice and maintained for more than 10 generations before being used in the experiments.

*P*. *flavifemur* adults were collected from the same paddy fields where *N*. *lugens* was collected and maintained on TN1 with *N*. *lugens* for 3 generations before being used in the bioassay.

### Tri-trophic bioassay with *P*. *flavifemur*

Newly emerged female and male *P*. *flavifemur* adults from TN1 rice were paired in a glass tube (Diameter 2 cm, Height 25 cm) that contained a 45-day-old Cry1C, Cry2A or non-Bt rice seedling in 10 mL Yoshida culture solution. Cotton wool was warped around the rice plants and sealed the glass tube to prevent insects escaping. After allowing 24 h for mating, twenty 3^rd^ instar *N*. *lugens* nymphs from the corresponding rice line were introduced to the wasps. After a 24 h exposure period, alive *N*. *lugens* nymphs were transferred into a new glass tube containing a corresponding rice seedling. Dead *N*. *lugens* nymphs were removed and checked under the microscope for signs of host feeding. The number of *N*. *lugens* killed by host feeding was recorded. Subsequently, a second batch of twenty *N*. *lugens* nymphs from the corresponding rice line was exposed to the same pair of *P*. *flavifemur* for another 24 h. Alive nymphs were transferred into a new glass tube and dead nymphs were counted in the same manner. New *N*. *lugens* nymphs were provided to *P*. *flavifemur* daily until the female wasp had died. Parasitized *N*. *lugens* nymphs were checked twice per day (9 am and 9 pm) and the time when parasitoids cocoons formed and adults emerged was recorded. Ten pairs of *P*. *flavifemur* were utilized for the Cry1C rice, Cry2A rice and non-Bt rice treatments. The offspring of *P*. *flavifemur* underwent another generation as described above. The developmental time, adult longevity and fecundity of *P*. *flavifemur* were estimated.

### Transfer of Cry1C and Cry2A through tri-trophic levels

An additional 10 pairs of *P*. *flavifemur* were set-up for each of the three rice lines parallel to the second generation study, as described above. For each treatment, five samples (replications) of the following materials were collected and analysed by ELISA: rice stem (100 mg per sample), *N*. *lugens* nymphs (3 insects pooled per sample), *P*. *flavifemur* larvae (10 larvae pooled per sample), *P*. *flavifemur* cocoons (10 cocoons pooled per sample), newly emerged male *P*. *flavifemur* (5 males pooled per sample), and newly emerged female *P*. *flavifemur* (5 females pooled per sample). In addition, groups of 5 female *P*. *flavifemur* were contained in a glass tube containing a rice seedling and *N*. *lugens* nymphs, and groups of 5 female *P*. *flavifemur* were contained in a glass tube containing a rice seedling only. In total, 5 glass tubes were set up for each treatment. After 48 h, five *N*. *lugens*-fed *P*. *flavifemur* samples (5 females pooled per sample) and five non-*N*. *lugens*-fed *P*. *flavifemur* samples (5 females pooled per sample) were collected for ELISA analyses. The Cry1C and Cry2A protein concentrations in rice and insect materials were measured by enzyme-linked immunosorbent assays (ELISA) using Cry1C detection kits and Cry2A detection kits from Envirologix (Portland, ME). Prior to analysis, all insects were washed with Phosphate Buffered Saline + Tween 20 (PBST) four times to remove any Bt toxin from the surface. Rice samples were diluted at a rate of 1:2000 (mg sample: µL PBST buffer) and fully ground by mortar and pestle. Insect samples were diluted at a rate of 1:10 (mg sample: µL PBST buffer) in 1.5 ml centrifuge tubes, and ground by hand using a plastic pestle. ELISA was performed according to the manufacturer’s instructions. The detection limit for the two Cry proteins was 1 ng/g.

### Field experiments

Cry1C rice (T1C-19), Cry2A rice (T2A-1) and non-Bt rice (MH63) were planted at the Jinhua Plant Protection Experimental Station (Jinhua) and the Zhejiang Middle Experimental Station (Dongyang) in 2013 at the restricted field testing site. The experiments were managed following the Implementation Regulations on Safety Assessment of Agricultural Genetically Modified Organisms issued by the Ministry of Agriculture of the People’s Republic of China. At Jinhua, rice seeds were sown on 25 June, and seedlings were transplanted on 25 July. At Dongyang, rice seeds were sown on 1 July, and seedlings were transplanted on 25 July. At both sites, the field was divided into nine experimental plots in a 3 (treatments: Cry1C rice, Cry2A and non-Bt rice) × 3 (replications) completely randomized design. Each experimental plot was 15 × 15 m. Each plot was bordered on all sides by a 50-cm-wide unplanted walkway. Seedlings were hand transplanted at one seedling per hill spaced 16.5 × 16.5 cm apart, and the entire experimental field was surrounded by five border rows of non-Bt plants (MH63). Normal cultural practices for growing rice, such as fertilization and irrigation, were followed during the course of the experiment, except that no pesticides were applied. A white porcelain plate (46 cm length × 36 cm width × 3.5 cm height), as described by^43^, was used to monitor the density of *N*. *lugens* and *P*. *flavifemur*. This plate is made of metal, and its surface is painted white. On each sampling date, 30 randomly selected hills were sampled in each plot. When sampling, the plate was held at a 45° angle to the ground, and a single hill was carefully grasped at the lower stem and then quickly bent into the plate. The sampled hill was beaten vigorously against the side of the plate for 4–5 s periods (about 13–15 beats). Subsequently, *N*. *lugens* and female *P*. *flavifemur* on the plate were counted immediately. Samples were taken in all plots on a 7–15 day schedule, beginning 1 month after transplanting until the rice reached full maturity. There were four sampling dates at both sites.

### Statistical analyses

Data on life table parameters of *P*. *flavifemur*, Bt protein residues in plants and insects, and were all analyzed using one-way ANOVA and Tukey’s multiple comparison tests. Field data were analyzed by repeated-measured ANOVA and Tukey’s multiple comparison tests. Before analysis, all percentage data were arcsine transformed, but untransformed means are presented. All statistical calculations were performed with SAS version 9.1 package.
